# Sources of Misinterpretation in the Input and Their Implications for Language Intervention With English-Speaking Children

**DOI:** 10.1044/2023_AJSLP-23-00016

**Published:** 2023-05-17

**Authors:** Laurence B. Leonard, Patricia Deevy, Shelley L. Bredin-Oja, Mariel Lee Schroeder

**Affiliations:** a Department of Speech, Language, and Hearing Sciences, Purdue University, West Lafayette, IN; b Department of Communication Sciences and Disorders, Minot State University, ND

## Abstract

**Purpose:**

In English and related languages, many preschool-age children with developmental language disorder (DLD) have difficulties using tense and agreement consistently. In this review article, we discuss two potential input-related sources of this difficulty and offer several possible strategies aimed at circumventing input obstacles.

**Method:**

We review a series of studies from English, supplemented by evidence from computational modeling and studies of other languages. Collectively, the studies show that instances of failures to express tense and agreement in DLD resemble portions of larger sentences in everyday input in which tense and agreement marking is appropriately absent. Furthermore, experimental studies show that children's use of tense and agreement can be swayed by manipulating details in fully grammatical input sentences.

**Results:**

The available evidence points to two particular sources of input that may contribute to tense and agreement inconsistency. One source is the appearance of subject + nonfinite verb sequences that appear in auxiliary-fronted questions (e.g., *Is [the girl running]? Does [the boy like popcorn]?*) and as dependent clauses in more complex sentences (e.g., *Help [her wash the dishes]*; *We saw [the frog hopping]*). The other source is the frequent appearance of bare stems in the input, whether nonfinite (e.g., *go* in *Make him go fast*) or finite (e.g., *go* in *I go, you go*).

**Conclusions:**

Although the likely sources of input are a natural part of the language that all children hear, procedures that alter the distribution of this input might be used in the early stages of intervention. Subsequent steps can incorporate more explicit comprehension and production techniques. A variety of suggestions are offered.

One of the hallmarks of developmental language disorder (DLD) in English during the preschool years is inconsistency in the use of tense and agreement morphemes. Along with correctly using morphemes such as present third-person –*s*, past tense –*ed,* irregular past, and both auxiliary and copula *BE* forms, children with DLD can also be heard producing utterances such as *The horse run fast*, *Mommy coming home soon*, and *Him draw this picture.* Often, children with DLD at the age of 5 years continue producing errors of this type, even though their same-age peers with typical language development have reached mastery levels in the use of these morphemes. During the preschool years, these differences between children with DLD and their peers not only show statistical significance at the group level; tests that assess the degree of use of these morphemes also show good diagnostic accuracy (e.g., Rice & Wexler, 2001). The magnitude of these verb morpheme difficulties can be further appreciated from multiple studies showing that 5-year-olds with DLD lag behind typically developing children who are as young as 3 years of age, even when factors such as mean length of utterance and verb inventories are taken into account (see review in Leonard, 2014).

Given these prominent weaknesses with tense and agreement morphemes, intervention procedures designed to assist children with these forms have appeared in the literature. These have included procedures making use of recasts (Camarata & Nelson, 1992) or a combination of recasts and focused stimulation (e.g., Leonard et al., 2004). Although most intervention approaches have relied on implicit learning on the part of the child, some recent approaches incorporate explicit teaching of grammatical morphemes into their protocols (e.g., Finestack, 2018; Smith-Lock et al., 2013). Both implicit and explicit approaches usually operate under the assumption that more frequent exposure to tense and agreement morphemes is a key factor in promoting gains.

Although there is little doubt about the difficulties that tense and agreement morphemes pose for children with DLD, there is no consensus on why these morphemes stand out as especially problematic (see Leonard, 2014, for a review of alternative explanations). Accounts have varied from assumed delays in the emergence of a biologically based linguistic principle (e.g., Rice & Wexler, 1996) to deep-rooted weaknesses in procedural memory that affect nonlinguistic as well as linguistic learning (e.g., Ullman & Pierpoint, 2005). Like DLD itself, there seems to be a genetic component to these particular weaknesses (Bishop et al., 2006), though the source may prove to be multifactorial. In principle, if we knew the reasons for these special difficulties, we might be able to shape our intervention procedures around the core problem, thus improving the outcomes for these children.

## The Lure of Subject + Nonfinite Verb Sequences and Bare Stems in the Input

In this review article, we explore implications for intervention if one assumes that the tense and agreement morpheme weaknesses of children with DLD can be traced to the children's misinterpretation of details in their language input. We begin with the assumption that children with DLD have generally weak language skills, but the profile of extraordinary difficulty with tense and agreement results from how this more general weakness interacts with the typology of the language being learned. English is a prime case, though we will touch on how this profile is altered when children are learning other types of languages.

We review two possible input sources of misinterpretation. The first concerns the appearance in the input of sentence-final subject + nonfinite verb sequences such as *The girl like puppies* and *The boy laughing*. The second involves the frequent appearance of “zero-marked” bare stem verbs in the input (e.g., *I play*, *you play*, *we play*, *they play*). For each source, we discuss evidence indicating that children with DLD make errors that can be attributed to the input. We then offer some possible details that might be incorporated into intervention that might reduce the degree to which these input factors contribute to the children's tense and agreement morpheme difficulties.

### Subject + Nonfinite Verb Sequences

Consider the following examples:

*The horse run fast*

*She buy a new car*

*Mommy coming home soon*

*A dog barking*

*The boy fix his bike*

*Her stop that*

*Her playing outside*

*Him draw this picture*

These utterances are clearly missing a tense and agreement morpheme, and three of them also reveal a pronoun error in subject position. However, these utterances, if taken as word sequences, represent propositions that children can, in fact, hear, as the following grammatical utterances reveal:

*Can [the horse run fast]?*

*Did [she buy a new car]?*

*Is [Mommy coming home soon]?*

*I hear [a dog barking]*

*Help [the boy fix his bike]*

*Make [her stop that]*

*We saw [her playing outside]*

*Watch [him draw this picture]*

In each of these grammatical utterances, a lexical verb (*run*, *buy*, *coming*, *barking*, *fix*, *stop*, *playing*, *draw*) is nonfinite because an element earlier in the sentence requires it. In the first three examples, a fronted modal auxiliary (*can*), auxiliary *DO* form (*did*), or auxiliary *BE* form (*is*) provides the agreement and/or tense information. In the remaining examples, a preceding lexical verb (*hear*, *help*, *make*, *saw*, *watch*) takes a nonfinite verb as its sentence complement.

To conclude that child utterances such as *The horse run fast* and *Mommy coming home soon* can be traced back to the input, it must be assumed that the children hear these sequences and fail to recognize that they are structurally tied to information (e.g., *can*, *is*, *help*, *saw*) that appears earlier in the input utterance. Without understanding these constraints, the children treat these sequences as appropriate for use as stand-alone utterances (see Tomasello, 2003). That is, these stand-alone utterances have the same status in the children's grammar as utterances such as *That frog hops* and *Daddy's working outside* that could have their basis in simple sentences heard in the input. Also, just as simple grammatical sentences in the input can then serve as a basis for children's own creations using the same constructions (e.g., from *That frog hops* to *This guy falls*), so too can inappropriately extracted nonfinite sequences serve as the basis for new (ungrammatical) creations by the child (e.g., from *The horse run fast* to *That cat purr*). There are several types of evidence that are consistent with this assumption.

First, consider sentence constructions in which there is a separation between a sentence element and its “interpreted” position, sometimes called “long-distance dependencies,” as in the following examples. We use the notation of underlining the element of interest and indicate its interpreted position with _____.

*Claudette was pushed _____ by Antonella.*

*Who was Lars pushing _____?*

*The car that the taxi hit _____ was blue.*

There is strong evidence that children with DLD have significant difficulties comprehending these types of long-distance dependencies. Examples of studies on passives include Hestvik et al. (2010), Montgomery and Evans (2009), and van der Lely (1996). For *wh*-object questions, examples include Deevy and Leonard (2004), Epstein et al. (2013), and van der Lely and Battell (2003). Evidence for DLD weaknesses in comprehending object relative clauses can be seen in the studies of Dick et al. (2004), Hestvik et al. (2022), and Montgomery et al. (2017).

Of course, the long-distance “dependencies” in *Did she buy a new car?* and *Help the boy fix his bike* are quite different from those seen in passives, *wh*-object questions, and object relative clauses. Yet, they share the property of requiring the learner to make use of earlier information when dealing with the later parts of the sentence. In cases such as passives, proper semantic interpretation is at stake. In cases such as *Did she buy a new car?* and *Help the boy fix his bike*, proper use of tense and agreement is in the balance. Although semantic interpretation may not be challenging in the *Did she buy…* or *Help the boy fix…* examples, the fact that these sentences contain nonfinite verbs that immediately follow their subjects could lure children into treating these subject + nonfinite verb sequences as acceptable. In fact, because these sequences represent meaningful propositions (*she buy a new car*; *the boy fix his bike*), the lure may be even greater.

There are several more direct sources of evidence for the appeal of subject + nonfinite verbs in the input. In studies of young children with typical language development (TLD) ages 2;6–3;0 (years;months), when children are still inconsistent in using tense and agreement morphemes, they tend to produce novel verbs in the same form in which they are consistently heard, even when the context changes to render the heard form ungrammatical. For example, Theakston et al. (2003) found that when the children heard the novel verb *mib* consistently in sentences such as *Will it mib?*, the children continued to produce *mib* rather than *mibs* when tested in the context of “What does this one do? It ___.” Yet, when a novel verb was consistently heard with the third-person singular –*s* inflection (e.g., *This one tams*), the children were much more likely to produce the inflection in the context, “What does this one do? It ___.” Similar findings were reported by Finneran and Leonard (2010).

This suggests that the development of tense and agreement use is built up in part by the interaction between the children's input and their developing ability to interpret this input. Computational models have attempted to simulate this effect. They begin by building into the model an utterance-final bias and initially restricting the learning span to mimic young children's limited processing ability (Croker et al., 2001; Freudenthal et al., 2006, 2007, 2009, 2010). When presented with transcripts of actual adult-to-child input, the output of the model shows the kinds of utterances illustrated above, including those with pronoun errors, as in *Her playing outside*. When the learning span of the model is gradually increased to reflect development, the proportion of subject + nonfinite verb sequences in the output decreases.

#### Input Effects in DLD

Thus far, we have discussed the plausibility of subject + nonfinite verb errors reflecting misinterpretations of the input and have referred to studies of young children with TLD. However, these children cease making such errors well before children with DLD. It needs to be shown that input effects are also implicated in the slower acquisition of tense and agreement morphology in children with DLD.

Several experimental studies point in this direction. Leonard and Deevy (2011) conducted a novel verb learning study with 4- and 5-year-olds with DLD and a group of same-age peers with TLD. The children with DLD were inconsistent in their use of tense and agreement morphemes, whereas the children in the TLD group were at mastery levels. Half the novel verbs were presented in nonfinite contexts only, as in *We saw the dog pagging*. The other half were heard only with auxiliary *was*, as in *Just now the horse was channing*. After the exposure period, the children's use of the novel verbs was tested in contexts requiring auxiliary *is* (e.g., “Tell me what's happening here”). The children with TLD used *is* with all novel verbs. In contrast, the children with DLD were more likely to use auxiliary *is* if the novel verbs had been heard in the auxiliary *was* context than in the nonfinite context. During testing, items were included that used different characters serving as the subjects of the sentences (e.g., a mouse rather than a dog *pagging*). Yet, these items yielded the same pattern of responses seen for items that used the same subjects as those used during the exposure period. This last point is important because it suggests that once a new verb is heard strictly in nonfinite form, it can be transferred to other utterances involving different subjects.

Leonard et al. (2015) conducted a novel verb learning study with 4-year-old children with DLD and a group of younger children with TLD matched for mean length of utterance. As is the case in such comparisons, the TLD group showed greater use of tense and agreement morphemes than the DLD group, yet the TLD group had not reached the level of mastery. Depending on the novel verb, nonfinite contexts used during the exposure period were of the type *Let's watch the dog fimm* and *Does the cat brack?* Finite contexts were of the type *All day long the dog kreffs* and *Do you think the cat swopes?* Testing after the exposure period included items requiring third-person singular –*s* (“Every day the cat ___”) and those requiring a nonfinite form (“We wanna watch the cat ___”). The children with TLD were influenced by the input context but not to the degree seen in the DLD group. An especially interesting finding was how the children with DLD showed more inappropriate productions of –*s* on nonfinite test items when the novel verbs had been presented in third-person singular form during the exposure phase. Such errors were of the type “We wanna watch the horse*…swopes*.”

A basic assumption behind input effects is that children showing inconsistent use of tense and agreement morphemes have not accurately sorted out contexts in which attested subject + nonfinite verb sequences are and are not appropriate to use. If this is true, then there should be evidence of some of this difficulty on comprehension measures. A second experiment by Leonard and Deevy (2011) examined this issue. Children with DLD at the ages of 4 and 5 years participated, as well as a group of 3-year-olds with TLD matched according to scores on a general comprehension test. The children's use of auxiliary *is* was first tested, which revealed greater proficiency on the part of the TLD group. A comprehension task was then administered requiring the children to point to the correct picture in response to sentences such as *The cow sees the horse eating*. Foil pictures depicted events such as a horse watching a cow eating and a horse eating while a cow is looking away. To ensure that the children understood the individual elements within these sentences, simple control sentences were also tested such as *The cow sees the horse* and *The horse is eating.* All children were near ceiling on the control sentences. However, on sentences of the type *The cow sees the horse eating*, the children with DLD were less accurate than their younger typically developing peers. Souto et al. (2016) replicated this finding with the same target structure but a slightly different set of foils.

One of the most likely sources of subject + nonfinite verb sequences is the appearance in the input of questions with fronted auxiliaries. Testing children's comprehension of these questions is not as straightforward because it is assumed that children hear the fronted auxiliaries and interpret the utterance as a question. What is at issue is whether the children understand the dependency connection between the fronted auxiliary and the information later in the question. Deevy and Leonard (2018) approached this issue through use of a looking-while-listening task. Children saw pairs of pictures on a screen such as a picture of a boy running and a picture of several dogs running. They then heard sentences such as *Are the nice little dogs running?* or *See the nice little dogs running?* For the first type of sentence, children could anticipate the picture of the dogs given the appearance of plural *are* at the beginning of the sentence. This could lead children to focus on the picture of the dogs before they actually hear the word *dogs*. On the other hand, *See the nice little dogs running?* provides no such opportunity for anticipatory looking. Deevy and Leonard found that younger TLD children (*M*_age_ = 3;6) began to focus their gaze on the proper picture before hearing the noun, whereas the DLD group (*M*_age_ = 5;11) did not show a clear pattern of gaze until they actually heard the noun. This finding suggests that the children in the TLD group were doing more than treating the fronted auxiliary as a pragmatic indicator of a question; they were actually treating the auxiliary as structurally related to other information to come in the sentence. The DLD group did not show evidence of this kind of understanding. Importantly, the children in the TLD group were already producing auxiliary *is* and *are* with over 90% accuracy, whereas the DLD group used *is* and *are* with 70% and 62% accuracy, respectively.

Recall that in the Leonard et al. (2015) study, children with DLD often produced third-person singular –*s* with verbs that had been heard strictly in this form even on subsequent test items requiring nonfinite verbs (as we saw with the example, “We wanna watch the horse*…swopes*”). This finding is in line with the assumption that the children did not understand the dependencies between earlier-appearing elements in the sentence and the type of verb form to use. In an investigation making use of electrophysiological evidence, Purdy et al. (2014) examined this issue with a group of school-age children with a history of DLD and a group of same-age children with TLD. The children heard fully grammatical sentences, as well as simple sentences with agreement commission errors (e.g., *Every day, the girls drives home*) and complex sentences with commission errors requiring the processing of long-distance relationships (e.g., *The dad watches the boy eats cookies*). The DLD group responded much like the TLD group when listening to simple sentences with agreement errors by showing a clear “P600” neural response. However, unlike the TLD group, the children with DLD showed less sensitivity to agreement commission errors in complex sentences. It seemed like the children with DLD were influenced by the local agreement (e.g., *the boy eats cookies*) reflected in the dependent clause.

The experimental evidence seems consistent with the idea that children with DLD have difficulty relating subject + nonfinite verb propositions to information appearing earlier in the input sentence (or, in the case of the above looking-while-listening study, vice versa). Freudenthal et al. (2021) conducted a computer simulation of this difficulty by building into the model a learning factor that controls the model's ability to associate elements in the sentence that occur in different time steps. This was operationalized by having the model predict the verb inflection in input utterances. Each word in the utterance was treated as occurring in a different time step. Sentence-level cues tied to words occurring earlier in the utterance had less predictive weight than cues occurring nearer to the inflection. For example, the pronoun *he* in *He runs* can serve as a cue to the third-person singular –*s* inflection. However, in the input utterance *Does he run?*, the pronoun *he* occurs closer in time to “run,” which attenuates the weight of the earlier occurring cue, “does.” As a result, *he run* (from *Does he run?*) competes with *he runs.* When Freudenthal et al. tested their model, the model's output showed slow but gradual learning of the third-person singular inflection. This pattern of learning was capturing the fact that, in English, nonfinite (bare stem) verb forms appear later in utterances. Freudenthal et al. then simulated learning by children with DLD by increasing the attenuation levels, which lowered the model's sequential learning abilities. The resulting output reflected the more protracted period of learning third-person singular –*s* seen in actual DLD data.

Can weaknesses in appreciating dependencies between tense and agreement morphemes and earlier-appearing material be improved through intervention? Fey et al. (2017) pursued this question in an intervention study aimed at facilitating use of auxiliary *is* and third-person singular –*s* in a group of children with DLD ages 3;3–4;7. The children were randomly assigned to either an experimental treatment or a more traditional treatment. For the experimental treatment, the children heard stories and received recasts that included the target morphemes but in strictly declarative contexts. They also participated in a comprehension component involving yes/no questions in which the correct answer depended on the tense of the fronted auxiliary. An example for auxiliary *is* treatment was *Is/was the boy diving into the pool?* For third-person singular –*s*, an example was *Does/did the boy see the dog?* Responding correctly depended on the child recognizing that the question referred to a past and not present event or vice versa. This component was designed to emphasize the relevance of the fronted auxiliary to the sentence as a whole—an insight that was hypothesized to be lacking in the children. The traditional approach also used focused stimulation and recasts, but half were in declarative form and half were in interrogative form. The comprehension component included the same questions used in the experimental treatment condition except that the children could respond correctly simply by understanding the agents and actions in the question (e.g., *Was the girl/boy diving into the pool?*). Clear effects of treatment were seen for the auxiliary *is* target. Specifically, the experimental condition was associated with greater gains in the children's production of auxiliary *is* in declarative position. However, the two types of treatment did not differ for the third-person singular target. Fey et al. noted that the children in the experimental condition made gains in comprehending the difference between *does* questions and *did* questions. They speculated that the minimal transfer to third-person singular –*s* production was because the children did not clearly relate the fronted auxiliary *does* to the present singular inflection –*s* (compare *Does the boy see the dog?* and *The boy sees the dog*). In contrast, this connection is more transparent for auxiliary *is* given the identical phonetic form in interrogative and declarative positions (as in *Is the girl climbing the ladder? The girl is climbing the ladder*).

Although subject + nonfinite verb utterances are frequently produced by English-speaking children with DLD during the preschool years, English is not the only language in which children with DLD show more extensive use of these kinds of utterances than their peers with TLD. In some of these languages, nonfinite productions come in the form of overt infinitive inflections in place of overt tense and agreement inflections. Swedish and Dutch are two such languages. Consider the following examples (we use “drink coffee” throughout our examples to facilitate translation):

Swedish: Lars dricka kaffe

    “Lars drink coffee”

    (Correct: Lars dricker kaffe)

    “Lars drinks coffee”

Dutch:  Anna koffie drinken

    “Anna coffee drink”

    (Correct: Anna drinkt koffie)

    “Anna drinks coffee”

In the Swedish example, −*a* in *dricka* “drink” is an infinitive inflection instead of the correct present tense –*er*. In the Dutch example, –*en* as in *drinken* “drink” is an infinitive inflection instead of the correct present tense –*t*. Also, in the Dutch example, we see the infinitive in sentence-final position. Let us look now at how questions with fronted modal auxiliaries are formed in these two languages:

Swedish: Kan [Lars dricka kaffe]?

     “Can [Lars drink coffee]?”

Dutch:  Kan [Anna koffie drinken]?

     “Can [Anna coffee drink]?”

From the Swedish example, it can be seen that Swedish resembles English in that a nonfinite verb is used when the modal auxiliary appears earlier in the sentence. In Swedish, of course, infinitives carry overt inflections rather than being bare stems as in English. However, in Dutch, when a fronted modal auxiliary is used, the infinitive (with its overt infinitive inflection) appears in sentence-final position. Therefore, the problem with *Anna koffie drinken* is not the location of the infinitive in the sentence, but rather the use of an infinitive instead of the present tense form when there is no accompanying auxiliary to express tense or agreement. If we assume the origins of the production came from misinterpreting the input, the utterance is not surprising. German shares with Dutch this same feature.

Subject + nonfinite verb productions occur in DLD in Swedish (e.g., Hansson et al., 2000), Dutch (e.g., de Jong, 2004), and German (e.g., Rice et al., 1997). However, they are not as frequent as in English. One possible reason is that whereas many questions are formed with an auxiliary *DO* in English, as in *Does Carol drink coffee?*, these other languages simply use the finite lexical verb in sentence-initial position, as in the Swedish *Dricker Lars kaffe?* (“Drinks Lars coffee?”).

In Romance languages such as Italian and Spanish, subject + nonfinite verb errors by children with DLD are even less frequent than in the languages just discussed and are described as quite uncharacteristic of these languages (e.g., Bedore & Leonard, 2001; Bortolini et al., 1997). It is probably no coincidence that sequences of this type in the input are not as common. There is no equivalent of the English auxiliary *DO* in questions. Instead, questions are often phrased with declarative word order, as in Italian *Gina beve caffè?* and Spanish *Sofía bebe café?* (“Gina/Sofía drinks coffee?”). Questions in English with the modal auxiliary *will* (e.g., *Will Gina drink coffee?*) can be produced with future tense forms (Italian *Gina berrà il caffè?*; Spanish *Sofía beberá café?*). Questions with the equivalent of the modal auxiliary *can* will often be constructed with the modal adjacent to the main verb, rather than separated by being placed in sentence-initial position. This is especially true in Italian (e.g., *Gina può bere il caffè?* “Gina can drink coffee?”). In short, these languages offer fewer opportunities for children to hear subject + nonfinite sequences.

The idea that these cross-linguistic differences in subject + nonfinite verb use are related to input effects finds support in computational modeling studies. For example, Freudenthal et al. (2007) found that the degree of nonfinite use in the model's output was greatest when the input was English, intermediate for Dutch and German input, and much more limited when the input was Spanish. (See Jourdain & Lahousse, 2021, for compatible evidence from young French-speaking children.) Further support can be found in the Freudenthal et al. (2021) computational model study that simulated DLD. Recall that when the model was run with English input, the output showed a prolonged period of learning the third-person singular form. However, when Spanish input was used, the effects were less dramatic. This was expected given that the tense and agreement differences between DLD and TLD groups are smaller in Spanish than in English (see review in Leonard, 2014).

## Defaulting to Bare Verb Stems

The appearance of subject + nonfinite verbs in the input may not be the only factor influencing children's failure to use tense and agreement inflections. In English, children may be influenced by the sheer frequency of bare verb stems in the input. Many of these are “zero-marked” finite forms (e.g., *I run*, *you run*, *we run*, *they run*). In a corpus study of British English, Räsänen et al. (2014) found that verbs most likely to be used by adults as bare stems (in appropriate contexts) were those most likely to lack third-person singular –*s* in obligatory contexts in the speech of young TLD children. This suggested to Räsänen et al. that bare stems might serve as a type of default form. The children were hearing correctly used bare stems, but by hearing them so frequently, the children adopted these stems as appropriate to use even in unattested third-person singular contexts.

Kueser et al. (2018) asked whether the same could be true for children with DLD. Instead of looking at bare stems, these investigators examined the degree to which children with DLD and younger children with TLD produced verbs marked for third-person singular –*s* in obligatory contexts. Kueser et al. then examined whether this use was related to the degree to which the same verbs appeared in third-person singular –*s* form in a large American English corpus of adult speech to children. As expected, the children with DLD were less likely than younger peers with TLD to produce third-person singular –*s* in obligatory contexts. However, the two groups were quite similar in producing third-person singular –*s* in accordance with the relative proportion of this inflection in the corpus. Or, put in defaulting terms, both groups were less likely to produce this inflection with verbs that were the most likely to appear as bare stems in the corpus.

In the computational modeling study of Freudenthal et al. (2021) described earlier, the feasibility of a defaulting factor was also examined. Specifically, Freudenthal et al. removed from the input those adult-to-child utterances that were most likely to contain subject + nonfinite verb sequences (e.g., auxiliary-fronted questions). For English, this manipulation showed an output that still revealed a slow rate of learning the third-person singular form. These results were attributed to the overall frequency of bare stems in the English input.

Subsequently, Freudenthal et al. (2023) created a dual-factor model in which the defaulting factor was formalized by converting each verb in a child's transcript to a single form (e.g., *drink* or *drinks* in English, *drinkt* or *drinken* in Dutch) if the verb showed a strongly dominant form in the input corpus. The defaulting factor was given greater weight in the case of DLD. Other details of the model (e.g., the right-to-left processing bias) functioned as in earlier models. The output of this dual-factor model showed even greater correspondence to actual data than previous models. Again, simulations for TLD and DLD showed the expected group differences. Cross-linguistic differences in the predicted direction were also seen. In this case, however, the degree of difference between English and the other languages provided an even closer match to actual child data. Yet, defaulting did not prove to be a sufficient explanation for the observed differences. Freudenthal et al. noted that the utterance-final learning bias built into the model was necessary along with the defaulting bias to produce the high levels of correspondence with the available child language evidence.

Although Spanish makes only limited use of subject + nonfinite verbs, in principle, children learning this language might resort to defaulting. For Spanish, the most likely default form would be the present tense third-person singular form as it is the most frequent in the language and represents the most frequent (though not the only) substitute used by children (see Aguado-Orea & Pine, 2015). In fact, Grinstead et al. (2013, 2018) have interpreted the children's frequent use of present third-person singular as constituting a type of nonfinite form. When Freudenthal et al. (2023) applied the defaulting factor to Spanish input in their model, defaulting appeared in the output to a more restricted degree than in the other languages, though third-person singular was, in fact, the most likely substitute.

The frequency difference between candidates for default use and unlikely candidates is much smaller in Spanish than in English. In English, bare stems appear throughout the paradigm, whereas third-person singular in Spanish competes with many other inflections. However, in the early stages of learning particular verbs, “competes” may be a misleading term. In a study of fast mapping, Bedore and Leonard (2000) found that Spanish-speaking 3-year-olds were more likely to recognize a novel Spanish-like verb that was consistently heard with the same inflection than a novel verb that varied in its inflections. In that study, the verb stems of the verbs occurred with the same frequency in both conditions; it was only the stem-inflection combinations that varied in frequency. Rather than “competing” with other inflected forms of the same verb, then, the more frequently occurring form of the verb may be recognized in the input more readily, possibly as even distinct from the same verb when it is used with other inflections.

## Language Learning Weakness Meets Language Typology

We have noted some examples of utterances from children with DLD that, on first appearance, seem quite peculiar, such as the English *Him draw this picture.* However, rather than reflecting an unnatural language learning mechanism, these examples could represent what happens when children with a broader based language deficit are dealing with a target language with particular typological characteristics.

Yet, counterintuitively, the diversity of errors across languages might actually be helpful in allowing us to better understand the nature of the broader deficit. The surface forms of *Him draw this picture* and *Anna koffie drinken* may be different from each other, but together, they implicate a problem connecting later appearing elements to early sentence elements. This problem, in turn, may suggest one source of the broader weakness in language. Take, for example, the proposal of McMurray et al. (2022) that children with DLD may have a weakness in inhibiting competing forms. When children with DLD are faced with sentences requiring an element to be related back to an earlier element in the sentence, they may have difficulty resisting the *semantically complete* nature of the subject + nonfinite sequence (e.g., *she buy a new car*; *Mommy coming home soon*; *him draw this picture*). That is, the semantically interpretable nature of this sequence may suppress the search for the separated element (e.g., *did*, *is*, *help*) that is responsible for the nonfinite form of the sequence in the first place. This underlying weakness might be universal in DLD but more likely to be manifested when a particular language makes significant use of sequences that, when separated from earlier elements in the sentence, are meaningful propositions that have the potential to be communicated as stand-alone utterances.

Also compatible with the notion of weaknesses with inhibition is the finding that children with DLD differ in their degree of defaulting as a function of the language being learned. Such a deficit would lead to the expectation that bare stems would dominate as the error forms in English because the high frequency of such forms in the input would make it a strong competitor in almost any sentence context. The present third-person singular form in Spanish would also be expected to be the most difficult for children with DLD to inhibit, though its lower relative frequency compared to bare stems in English would result in a less dramatic case of defaulting. The potential for defaulting might be universal, but its conspicuous use by children with DLD could be dictated by the presence and strength of competing forms in the language.

The McMurray et al. (2022) proposal is surely not the only one that might be pursued to gain a better understanding of DLD. Our point is to show that alternative explanations for DLD might be refined or even discarded based on whether they offer a reasonable account of how input effects can shape the grammatical profiles of the children.

## Implications for Intervention

Although much work remains to determine how input interacts with the broader language deficits seen in DLD, the evidence accumulated thus far provides some potential directions for intervention. Several examples follow. All are based on the assumption that children's difficulties are not likely due to faulty input from parents or others but rather to limitations in the children's intake and interpretation of the input. By altering the distribution of particular types of forms in the input, clinicians (ideally in collaboration with parents) might be able to help children develop the insights needed to gain greater consistency in tense and agreement use.

Our examples serve as suggestions intended to augment rather than replace current evidence-based practices. Many useful findings have emerged from the literature on ways to facilitate tense and agreement use in children with DLD. Recent examples include using imitation primarily to allow children to obtain early production success in intervention rather than as a long-term procedure (see Eisenberg et al., 2020) and, for past tense treatment, focusing on verbs that are (counterintuitively) atelic, relatively low in frequency, and more phonologically complex (Owen Van Horne et al., 2018). Our concern is that, even when children's ability to produce tense and agreement forms becomes stronger with the help of such procedures, the children may still lack the awareness of when these forms must be produced. We believe that this awareness might be fostered through input manipulations and activities that promote children's awareness of differences in input structures.

### Reducing the Impact of Subject + Nonfinite Verb Sequences

The use of auxiliary-fronted questions is a central part of English. Unfortunately, before children have recognized the structural links between the auxiliary and the later portions of the utterance, there is the risk that the later-appearing subject + nonfinite verb sequence takes hold as a basis for generating new utterances. This presents a dilemma for practitioners because whereas questions are important to teach, they are also a potential source of continued use of nonfinite verbs on the part of the child.

Paradoxically, just the opposite might be assumed—that auxiliary-fronted questions would be an excellent way to introduce and teach auxiliary forms given their seemingly salient sentence-initial position. Yet, a study by Fey and Loeb (2002) illustrates the potential pitfalls in taking this view. Fey and Loeb asked whether the use of recasts with auxiliary-fronted auxiliary *is* questions (e.g., *Is that man eating a cookie?*) and auxiliary *will* questions (e.g., *Will that boy fall?*) would assist young children with DLD in acquiring these particular auxiliaries or, more broadly, auxiliaries in general. At the outset of the study, the children were not yet using auxiliaries in their own utterances. Unfortunately, treatment was unsuccessful: The children's gains in using both the target morphemes and the broader class of auxiliary *BE* and modal auxiliaries were no greater than the gains seen by a comparison play group that was not provided recasts. In fact, for auxiliary *is*, there was a trend for the (modest) gains to be higher in the play group than the group receiving the auxiliary-fronted recasts. It appears that the fronting of the auxiliaries had no particular impact on the children's language and, worse, might have given the children more opportunities to conclude that nonfinite verbs can directly follow subjects (*that man eating a cookie*; *that boy fall*).

One possible alternative would be to postpone targeting auxiliary-fronted questions until the children have acquired some skill with the declarative counterparts of the questions. For questions with auxiliary *BE* and modal auxiliaries, this seems relatively straightforward (e.g., *Mommy is going outside*; *That horse can run really fast*). As a next step, activities might pair declaratives with auxiliaries and auxiliary-fronted interrogative versions of the same sentences (e.g., *The bus is going fast* – *Is the bus going fast?*). When presented together in contexts that are compatible with how declaratives versus interrogatives are used, the nonfinite sequence (*the bus going fast*) might become more closely associated with fronted auxiliaries and no longer regarded as an acceptable alternative in declarative contexts. The temporally close pairing of the declarative and interrogative equivalents is likely to be important. If the declarative and interrogative versions are separated in time, the input might approximate children's usual input. Recall that a basic assumption is that one reason for children's inconsistency is that they hear in the input both declarative sentences with the auxiliary adjacent to the main verb (e.g., *Angie is going home now*) and similar questions with the auxiliary separated from the main verb (e.g., *Is Angie playing outside?*), which can provide the basis for nonfinite use (e.g., *Angie going outside*). As a result, both the with-auxiliary and without-auxiliary versions have the same communicative status in the children's grammar. The close temporal pairing of the declarative and interrogative versions might help the child recognize that declaratives always have the auxiliary.

Unfortunately, the structural relationship between questions with auxiliary *DO* and the corresponding declaratives is opaque (*does the girl like ice cream – the girl likes ice cream*; *did the boy wash the car – the boy washed the car*). As we saw in the Fey et al. (2017) treatment study, children do not seem to recognize this relationship as readily as the relationship between declaratives and questions with auxiliary *BE* forms, as in *The bus is going fast* – *Is the bus going fast?* Employing declaratives with auxiliary *DO* could be appropriate if the pragmatic context is altered to involve agreeing with a previous assertion (as in *Does the girl like ice cream? Yes, the girl does like ice cream*). However, it is not clear if such an activity would have any effect on children's use of tense and agreement in more typical declarative sentences (such as *The girl likes ice cream*).

The Fey et al. (2017) study was much more successful in finding a way to emphasize the relationship between fronted auxiliary *BE* forms and the later appearing subject + nonfinite verb sequences. Recall that these investigators required the children to respond to questions in which the correct answer depended on the tense of the fronted auxiliary (e.g., *Is/was the girl climbing the ladder?*). Treatment activities that included this component were associated with significant gains in the children's use of auxiliary *BE* in declaratives. A similar strategy might be used for contrasts such as *Is/are the fish jumping?* By having a singular/plural as well as a present/past contrast, the relevance of the sentence-initial auxiliary might become clearer. However, children's awareness of the invariant number in words such as *fish*, *deer*, and *moose* would be required to ensure that responses to the *is/are* items relied on attention to the auxiliary and not to the cues provided by overt singular/plural differences in the noun (as would be the case in *Is the girl jumping?* vs. *Are the girls jumping?*).

Questions are often used to engage children in conversation, and there are likely many contexts in which alternative ways to elicit responses could be just as effective without using subject + nonfinite sequences. For example, instead of *Does this kind of dinosaur eat grass?*, the alternative *I wonder if this kind of dinosaur eats grass* might be used. Note that the child might not know that *wonder if* requires a finite verb in the sentence complement; the point is that the sentence complement (*this kind of dinosaur eats grass*) will not lead the child astray.

Constructions with nonfinite dependent clauses (e.g., *Make that horse jump*; *We watched Sarah run the race*) are another possible source of children's subject + nonfinite verb utterances. Early in treatment, such constructions might well be avoided altogether, especially if the children's comprehension of complex syntax is in doubt. Constructions with nonfinite dependent clauses are not as frequent in the input as questions and therefore may play a smaller role in children's nonfinite verb use. However, they may play an outsize role in contributing to children's use of utterances with pronoun errors such as *Me open this* (from *Help me open this*) and *Her take my car* (from *I saw her take my car*). One potential way to reduce children's use of nonfinite dependent clauses as separate utterances might be to present pairs such as *We saw her playing outside. She was playing outside.* Pairs of this type might more closely associate the nonfinite clause (and pronoun forms such as *me* and *her*) with preceding material in the same sentence.

When teaching sentences with dependent clauses of this type, it might prove helpful to begin with nouns rather than pronouns immediately preceding the nonfinite verb (e.g., *We saw the girl playing outside* rather than *We saw her playing outside*). Imagine a modeling procedure in which the child observes the clinician and a model (a person or puppet) in a prearranged dialogue. An utterance by the clinician could be followed by an utterance by the model and then the reverse for the next pair of utterances. In this way, the child could hear a simple finite sentence and a similar sentence with an embedded subject + nonfinite verb. Examples could include:

Clinician: *Let's watch the horse eat hay.*

Model: *Every day the horse eats hay.*

Model: *Let's watch the bird eat worms.*

Clinician: *Every day the bird eats worms.*

Following several pairs of utterances presented in this way, the child could replace the model in attempting both types of utterances. Once the types of sentences requiring a finite versus nonfinite verb form become clearer to the child, similar sentences involving pronouns might be introduced.

There is renewed interest in treatment approaches that involve explicit instruction to assist children's grammatical abilities (e.g., Balthazar et al., 2020; Finestack, 2018). Because there is only a limited number of matrix verbs that call for nonfinite verbs in dependent clauses, explicit teaching approaches might be most appropriate. Much like teaching which verbs are irregular in past tense, practitioners might have to teach specific matrix verb–nonfinite clause constructions on a one-by-one basis. In some instances, should children's metalinguistic abilities allow for it, distinctions might be made such as the fact that some “perception” verbs take nonfinite dependent clauses (e.g., *We heard her playing the piano*; *I saw him break the window*), while “cognition” verbs do not have that option (e.g., *We think she was playing the piano*; *I know he broke the window*).

### Reducing the Effects of Defaulting

Subject + nonfinite verb sequences in larger structures may not be the only source of children's use of nonfinite verb forms in contexts requiring tense and agreement marking. Especially in English, bare stems abound in the input. Many of these are “zero-marked” finite forms (e.g., *I play*, *they go*, *we sleep*). Although zero-marked finite forms do not appear with third-person singular subjects, their omnipresence makes them easy substitutes when children are still inconsistent with tense and agreement forms.

Defaulting to bare stems can occur at two levels. At a more general level, the overall frequency of bare stems in the input can lead children to adopt bare stems as the form of choice across the verbs they use. At a more specific level, some verbs may appear in the input in bare-stem form more frequently than other verbs. Those with high bare stem frequency might be more likely to be used as bare stems in contexts requiring overt tense and agreement forms. We will consider the general- and specific-level cases in turn.

Procedures to counteract children's use of bare stems across verbs in general are not likely to differ from prevailing approaches in the clinical literature. Those approaches identified at the outset of this review article are likely to be appropriate. These include conversational recasting, focused stimulation, auditory bombardment, and others that provide an increase in the frequency of verbs overtly marked for tense and agreement. Some of these approaches target specific morphemes, whereas others have as their aim greater exposure across a wider variety of tense and agreement forms. These approaches do not necessarily assume that input factors are the cause of the grammatical difficulty, though they do share the view that enhancing exposure to tense and agreement forms can be beneficial to the children.

An example of the latter is “toy talk”—an approach first designed to assist parents in their interactions with their children (e.g., Hadley et al., 2011; Hadley & Walsh, 2014). In this approach, tense and agreement morphemes are viewed as a constellation of related forms (see Rispoli et al., 2009, 2012). In toy talk, the adult interacts with the child and focuses on comments about the actions of toy characters and other objects during play. This emphasis results in a naturally occurring increase in the degree to which overt tense and agreement forms are used.

Also, at a more general level, explicit tactics might be incorporated, even within approaches that are ordinarily viewed as implicit (see Baron & Arbel, 2022). For example, Leonard et al. (2004) used a focused stimulation procedure to help children with DLD acquire tense and agreement morphemes. They reasoned that although the stories they created provided multiple examples of appropriate tense and agreement use, these stories provided children with no indication that the alternative subject + nonfinite verb utterances were *not* appropriate. Accordingly, in each story, these researchers built in an exchange in which one of the characters produced a subject + nonfinite verb utterance and then explicitly self-corrected, as in: “Do you know where Bobby's grandmother lives? She live on a farm. Whoops, I meant to say she *lives* on a farm!” The contribution of this cue could not be separated from the other elements of the treatment package, though, overall, children with DLD made reliable gains on tense and agreement morphemes relative to gains on control forms.

At a more specific level, experimental studies of input effects have shown that children are prone to use a novel verb in the form in which it was most frequently heard. For example, the form *kreffs* might be used if it was consistently heard in a third-person context, but *kreff* might be the form adopted if the verb was consistently heard in a nonfinite context. Even if the child is later presented with a third-person singular context such as “Every day the girl ___,” the child will be more likely to use *kreff* instead of *kreffs* if only *kreff* had been heard in the input. This suggests strongly that it is not only the proportion of subject + third-person singular verb or subject + nonfinite verb sequences that are influential, but the specific verb used in these sequences. This specific-verb effect means that it may not be enough to help children use a tense and agreement morpheme with only select verbs. The morpheme may become too closely associated with these particular verbs, and thus, the children may continue to show spotty use of the morpheme when other verbs are required.

Thanks to studies conducted by Plante and her colleagues (e.g., Plante et al., 2014), there is a remedy for this potential problem. Plante et al. used conversational recasting to assist 4- and 5-year-old children with DLD in their acquisition of grammatical morphemes. For most children, these were tense and agreement morphemes. These investigators found that strong treatment effects occurred when the target morpheme was used with 24 unique verbs during recasting in each session. These gains included the children using the target morphemes with verbs that were not presented during treatment. A similar approach using fewer unique verbs with the target morphemes was not successful in leading to generalization.

Following Plante et al. (2014), a good first step toward promoting generalization might be to employ a wide range of different verbs in treatment for tense and agreement morphemes. This could increase the number of verbs that could be rebalanced if the children's input history with some of these verbs almost exclusively involved bare stems.

The implications for intervention for children speaking Spanish are somewhat different from those for English. As noted earlier, the rich inflection paradigm of Spanish and its use of finite lexical verbs in questions where English would employ auxiliary *DO* substantially reduce the instances of subject + nonfinite verb sequences in the input. However, defaulting can occur; in the case of Spanish, it would be children's use of the more frequent third-person singular form as a substitute rather than a nonfinite form. As a safeguard against children relying on third-person singular forms of the verb, clinicians might endeavor to teach several inflections with each new verb that is introduced in therapy. Probably not all inflections with the verb need to be required in the children's productions in the early stages, but exposures to more than one inflection for each verb should probably occur.

## Summary

Professionals providing services to English-speaking preschoolers with DLD are well acquainted with the slow development of tense and agreement forms in these children. Existing treatment efforts have clearly had some success, though gains in the children's skills have been hard-won. Most procedures provide ample examples of how tense and agreement forms should be used. However, there are contexts in English in which tense and agreement forms are not used, and these are in abundant display in children's everyday lives. It may not be clear to children why these forms are not just as appropriate to use in contexts that can alternatively be marked with tense and agreement. In this review article, we have pointed out details in natural input that might lead children with DLD to misinterpret the conditions in which tense and agreement forms can be disregarded. Treatment solutions to increase children's awareness of these conditions will probably require steps that supplement our usual practices. We have offered a variety of suggestions here in the hope they will prompt further study in this important area.

## References

[bib1] Aguado-Orea, J., & Pine, J. (2015). Comparing different models of the development of verb inflection in early child Spanish. PLOS ONE, 10 3, Article e0119613. 10.1371/journal.pone.0119613 25760035 PMC4356619

[bib2] Balthazar, C., Ebbels, S., & Zwitserlood, R. (2020). Explicit grammatical intervention for developmental language disorder: Three approaches. Language, Speech, and Hearing Services in Schools, 51(2), 226–246. 10.1044/2019_LSHSS-19-00046 32255746 PMC7225018

[bib3] Baron, L., & Arbel, Y. (2022). An implicit–explicit framework for intervention methods in developmental language disorder. American Journal of Speech-Language Pathology, 31(4), 1557–1573. 10.1044/2022_AJSLP-21-00172 35446629 PMC9531931

[bib4] Bedore, L., & Leonard, L. (2000). The effects of inflectional variation on fast mapping of verbs in English and Spanish. Journal of Speech, Language, and Hearing Research, 43(1), 21–33. 10.1044/jslhr.4301.21 10668650

[bib5] Bedore, L, & Leonard, L. (2001). Grammatical morphology deficits in Spanish-speaking children with specific language impairment. Journal of Speech, Language, and Hearing Research, 44(4), 905–924. 10.1044/1092-4388(2001/072) 11521782

[bib6] Bishop, D. V. M., Adams, C. V., & Norbury, C. F. (2006). Distinct genetic influences on grammar and phonological short-term memory deficits: Evidence from 6-year-old twins. Genes, Brain & Behavior, 5(2), 158–169. 10.1111/j.1601-183X.2005.00148.x 16507007

[bib7] Bortolini, U., Caselli, M. C., & Leonard, L. B. (1997). Grammatical deficits in Italian-speaking children with specific language impairment. Journal of Speech, Language, and Hearing Research, 40(4), 809–820. 10.1044/jslhr.4004.809 9263945

[bib8] Camarata, S. M., & Nelson, K. E. (1992). Treatment efficiency as a function of target selection in the remediation of child language disorders. Clinical Linguistics and Phonetics, 6(3), 167–178. 10.3109/02699209208985528 21269156

[bib9] Croker, S., Pine, J. M., & Gobet, F. (2001). Modelling children's case marking errors with MOSAIC. In E. Altmann, A. Cleeremans, C. Schunn, & W. Gray (Eds.), Proceedings of the Fourth International Conference on Cognitive Modeling (pp. 55–60). Lawrence Erlbaum.

[bib11] Deevy, P., & Leonard, L. B. (2004). The comprehension of *Wh*-questions in children with specific language impairment. Journal of Speech, Language, and Hearing Research, 47(4), 802–815. 10.1044/1092-4388(2004/060) 15324287

[bib12] Deevy, P., & Leonard, L. (2018). Sensitivity to morphosyntactic information in preschool children with and without developmental language disorder: A follow-up study. Journal of Speech, Language, and Hearing Research, 61(12), 3064–3074. 10.1044/2018_JSLHR-L-18-0038 PMC6440306 30453333

[bib10] de Jong, J. (2004). Grammatical impairment: An overview and a sketch of Dutch. In L. Verhoeven & H. van Balkom (Eds.), Classification of developmental language disorders (pp. 261–281). Lawrence Erlbaum.

[bib13] Dick, F., Wulfeck, B., Krupa-Kwiatkowski, M., & Bates, E. (2004). The development of complex sentence interpretation in typically developing children compared with children with specific language impairments or early unilateral focal lesions. Developmental Science, 7(3), 360–377. 10.1111/j.1467-7687.2004.00353.x 15595375

[bib14] Eisenberg, S. L., Bredin-Oja, S. L., & Crumrine, K. (2020). Use of imitation training for targeting grammar: A narrative review. Language, Speech, and Hearing Services in Schools, 51(2), 205–225. 10.1044/2019_LSHSS-19-00024 32255747

[bib15] Epstein, B., Hestvik, A., Shafer, V. L., & Schwartz, R. G. (2013). ERPs reveal atypical processing of subject versus object *Wh*-questions in children with specific language impairment. International Journal of Language & Communication Disorders, 48(4), 351–365. 10.1111/1460-6984.12009 23889832 PMC3728699

[bib16] Fey, M., Leonard, L., Bredin-Oja, S., & Deevy, P. (2017). A clinical evaluation of the competing sources of input hypothesis. Journal of Speech, Language, and Hearing Research, 60(1), 104–120. 10.1044/2016_JSLHR-L-15-0448 PMC5533554 28114610

[bib17] Fey, M., & Loeb, D. (2002). An evaluation of the facilitative effects of inverted yes-no questions on the acquisition of auxiliary verbs. Journal of Speech, Language, and Hearing Research, 45(1), 160–174. 10.1044/1092-4388(2002/012) 14748646

[bib18] Finestack, L. H. (2018). Evaluation of an explicit intervention to teach novel grammatical forms to children with developmental language disorder. Journal of Speech, Language, and Hearing Research, 61(8), 2062–2075. 10.1044/2018_JSLHR-L-17-0339 PMC6198921 30046804

[bib19] Finneran, D. A., & Leonard, L. B. (2010). Role of linguistic input in third-person singular –*s* use in the speech of young children. Journal of Speech, Language, and Hearing Research, 53(4), 1065–1074. 10.1044/1092-4388(2009/09-0056) PMC7251330 20605939

[bib20] Freudenthal, D., Gobet, F., & Pine, J. M. (2023). MOSAIC+: A crosslinguistic model of verb-marking errors in typically developing children and children with developmental language disorder. Language Learning. Advance online publication. 10.1111/lang.12580 36814394

[bib21] Freudenthal, D., Pine, J. M., Aguado-Orea, J., & Gobet, F. (2007). Modeling the developmental patterning of finiteness marking in English, Dutch, German, and Spanish using MOSAIC. Cognitive Science, 31(2), 311–341. 10.1080/15326900701221454 21635299

[bib22] Freudenthal, D., Pine, J. M., & Gobet, F. (2006). Modeling the development of children's use of optional infinitives in Dutch and English using MOSAIC. Cognitive Science, 30(2), 277–310. 10.1207/s15516709cog0000_47 21702816

[bib23] Freudenthal, D., Pine, J. M., & Gobet, F. (2009). Simulating the referential properties of Dutch, German, and English root infinitives in MOSAIC. Language Learning and Development, 5(1), 1–29. 10.1080/15475440802502437

[bib24] Freudenthal, D., Pine, J. M., & Gobet, F. (2010). Explaining quantitative variation in the rate of Optional Infinitive errors across languages: A comparison of MOSAIC and the Variational Learning Model. Journal of Child Language, 37(3), 643–669. 10.1017/S0305000909990523 20334719

[bib25] Freudenthal, D., Ramscar, M., Leonard, L., & Pine, J. (2021). Simulating the acquisition of verb inflection in typically developing children and children with developmental language disorder in English and Spanish. Cognitive Science, 45(3), Article e12945. 10.1111/cogs.12945 33682196

[bib26] Grinstead, J., Baron, A., Vega-Mendoza, M., De la Mora, J., Cantú-Sánchez, M., & Flores, B. (2013). Tense marking and spontaneous speech measures in Spanish specific language impairment: A discriminant function analysis. Journal of Speech, Language, and Hearing Research, 56(1), 352–363. 10.1044/1092-4388(2012/11-0289) 22992707

[bib27] Grinstead, J., Vega-Mendoza, M., & Goodall, G. (2018). Inversion and finiteness in Spanish and English: Developmental evidence from the optional infinitive and optional inversion stages. Language, 94(3), 575–610. 10.1353/lan.2018.0036

[bib28] Hadley, P. A., Rispoli, M., Fitzgerald, C., & Bahnsen, A. (2011). Predictors of morphosyntactic growth in typically developing toddlers: Contributions of parent input and child sex. Journal of Speech, Language, and Hearing Research, 54(2), 549–566. 10.1044/1092-4388(2010/09-0216) 20719872

[bib29] Hadley, P. A., & Walsh, K. M. (2014). Toy talk: Simple strategies to create richer grammatical input. Language, Speech, and Hearing Services in Schools, 45(3), 159–172. 10.1044/2014_LSHSS-13-0055 24687135

[bib30] Hansson, K., Nettelbladt, U., & Leonard, L. (2000). Specific language impairment in Swedish: The status of verb morphology and word order. Journal of Speech, Language, and Hearing Research, 43(4), 848–864. 10.1044/jslhr.4304.848 11386473

[bib32] Hestvik, A., Epstein, B., Schwartz, R. G., & Shafer, V. L. (2022). Developmental language disorder as syntactic prediction impairment. Frontiers in Communication, 6, 637585. 10.3389/fcomm.2021.637585 35237682 PMC8887879

[bib31] Hestvik, A., Schwartz, R., & Tornyova, L. (2010). Relative clause gap-filling in children with specific language impairment. Journal of Psycholinguistic Research, 39(5), 443–456. 10.1007/s10936-010-9151-1 20549559 PMC3017297

[bib33] Jourdain, M., & Lahousse, K. (2021). The development of constructions from the right edge: A multinomial regression analysis of clitic left and right dislocation in child French. Journal of Child Language, 48(5), 1023–1047. 10.1017/S0305000920000513 33143785

[bib34] Kueser, J. B., Leonard, L. B., & Deevy, P. (2018). Third person singular -*s* in typical development and specific language impairment: Input and neighbourhood density. Clinical Linguistics & Phonetics, 32(3), 232–248. 10.1080/02699206.2017.1342695 28727489 PMC6086116

[bib35] Leonard, L. B. (2014). Children with specific language impairment (2nd ed.). MIT Press. 10.7551/mitpress/9152.001.0001

[bib36] Leonard, L. B., Camarata, S., Brown, B., & Camarata, M. (2004). Tense and agreement in the speech of children with specific language impairment: Patterns of generalization through intervention. Journal of Speech, Language, and Hearing Research, 47(6), 1363–1379. 10.1044/1092-4388(2004/102) 15842016

[bib37] Leonard, L. B., & Deevy, P. (2011). Input distribution influences degree of auxiliary use by children with specific language impairment. Cognitive Linguistics, 22(2), 247–273. 10.1515/cogl.2011.010 23750074 PMC3673736

[bib38] Leonard, L. B., Fey, M. E., Deevy, P., & Bredin-Oja, S. L. (2015). Input sources of third-person singular –*s* inconsistency in children with and without specific language impairment. Journal of Child Language, 42(4), 786–820. 10.1017/S0305000914000397 25076070 PMC4430448

[bib39] McMurray, B., Apfelbaum, K. S., & Tomblin, J. B. (2022). The slow development of real-time processing: Spoken word recognition as a crucible for new thinking about language acquisition and language disorders. Current Directions in Psychological Science, 31(4), 305–315. 10.1177/09637214221078325 37663784 PMC10473872

[bib40] Montgomery, J. W., & Evans, J. L. (2009). Complex sentence comprehension and working memory in children with specific language impairment. Journal of Speech, Language, and Hearing Research, 52(2), 269–288. 10.1044/1092-4388(2008/07-0116) PMC4684953 18723601

[bib41] Montgomery, J. W., Gillam, R. B., Evans, J. L., & Sergeev, A. V. (2017). “Whatdunit?” Sentence comprehension abilities of children with SLI: Sensitivity to word order in canonical and noncanonical structures. Journal of Speech, Language, and Hearing Research, 60(9), 2603–2618. 10.1044/2017_JSLHR-L-17-0025 PMC5831622 28832884

[bib42] Owen Van Horne, A. J., Curran, M., Larson, C., & Fey, M. E. (2018). Effects of a complexity-based approach on generalization of past tense -*ed* and related morphemes. Language, Speech, and Hearing Services in Schools, 49(3S), 681–693. 10.1044/2018_LSHSS-STLT1-17-0142 30120446 PMC6198913

[bib43] Plante, E., Ogilvie, T., Vance, R., Aguilar, J. M., Dailey, N. S., Meyer, C., Lieser, A. M., & Burton, R. (2014). Variability in the language input to children enhances learning in a treatment context. American Journal of Speech-Language Pathology, 23(4), 530–545. 10.1044/2014_AJSLP-13-0038 24700145

[bib44] Purdy, J. D., Leonard, L. B., Weber-Fox, C., & Kaganovich, N. (2014). Decreased sensitivity to long-distance dependencies in children with a history of specific language impairment: Electrophysiological evidence. Journal of Speech, Language, and Hearing Research, 57(3), 1040–1059. 10.1044/2014_JSLHR-L-13-0176 PMC4433008 24686983

[bib45] Räsänen, S. H., Ambridge, B., & Pine, J. M. (2014). Infinitives or bare stems? Are English-speaking children defaulting to the highest-frequency form? Journal of Child Language, 41(4), 756–779. 10.1017/S0305000913000159 23830201

[bib46] Rice, M. L., Noll, K. R., & Grimm, H. (1997). An extended optional infinitive stage in German-speaking children with specific language impairment. Language Acquisition, 6(4), 255–295. 10.1207/s15327817la0604_1

[bib47] Rice, M. L., & Wexler, K. (1996). Toward tense as a clinical marker of specific language impairment in English-speaking children. Journal of Speech and Hearing Research, 39(6), 1239–1257. 10.1044/jshr.3906.1239 8959609

[bib48] Rice, M. L., & Wexler, K. (2001). Rice/Wexler Test of Early Grammatical Impairment. The Psychological Corporation.

[bib49] Rispoli, M., Hadley, P. A., & Holt, J. K. (2009). The growth of tense productivity. Journal of Speech, Language, and Hearing Research, 52(4), 930–944. 10.1044/1092-4388(2009/08-0079) 19641077

[bib50] Rispoli, M., Hadley, P. A., & Holt, J. K. (2012). Sequence and system in the acquisition of tense and agreement. Journal of Speech, Language, and Hearing Research, 55(4), 1007–1021. 10.1044/1092-4388(2011/10-0272) 22232389

[bib51] Smith-Lock, K. M., Leitão, S., Lambert, L., & Nickels, L. (2013). Effective intervention for expressive grammar in children with specific language impairment. International Journal of Language & Communication Disorders, 48(3), 265–282. 10.1111/1460-6984.12003 23650884

[bib52] Souto, S. M., Leonard, L. B., Deevy, P., Fey, M. E., & Bredin-Oja, S. L. (2016). Subordinate clause comprehension and tense/agreement inconsistency in children with specific language impairment. Journal of Communication Disorders, 6(2), 45–53. 10.1016/j.jcomdis.2016.05.002 27235928

[bib53] Theakston, A. L., Lieven, E. V. M., & Tomasello, M. (2003). The role of the input in the acquisition of third-person singular verbs in English. Journal of Speech, Language, and Hearing Research, 46(4), 863–877. 10.1044/1092-4388(2003/067) 12959465

[bib54] Tomasello, M. (2003). Constructing a language: A usage-based theory of language acquisition. Harvard University Press.

[bib55] Ullman, M. T., & Pierpoint, E. I. (2005). Specific language impairment is not specific to language: The procedural deficit hypothesis. Cortex, 41(3), 399–433. 10.1016/S0010-9452(08)70276-4 15871604

[bib56] van der Lely, H. (1996). Specifically language impaired and normally developing children: Verbal passive vs. adjectival passive sentence interpretation. Lingua, 98(4), 243–272. 10.1016/0024-3841(95)00044-5

[bib57] van der Lely, H., & Battell, J. (2003). Wh-movement in children with grammatical SLI: A test of the RDDR hypothesis. Language, 79(1), 153–181. 10.1353/lan.2003.0089

